# Pulled versus Pushed Monocanalicular Silicone Intubation in Adults with Lacrimal Drainage System Stenosis: A Comparative Case Series

**DOI:** 10.1155/2021/5592039

**Published:** 2021-09-01

**Authors:** Abolfazl Kasaee, Bahram Eshraghi, Kambiz Ameli, Hossein Ghahvehchian, Mansooreh Jamshidian-Tehrani, Amin Nabavi, Bahman Inanloo

**Affiliations:** ^1^Eye Research Center, Farabi Eye Hospital, Tehran University of Medical Sciences, Tehran, Iran; ^2^Eye and Skull Base Research Centers, The Five Senses Institute, Rassoul Akram Hospital, Iran University of Medical Sciences, Tehran, Iran; ^3^Department of Ophthalmology, Guilan University of Medical Sciences, Rasht, Iran

## Abstract

**Purpose:**

To compare the success rate and complications of pulled versus pushed monocanalicular intubation in adults with incomplete lacrimal drainage system obstruction (lacrimal drainage system stenosis).

**Methods:**

Patients with lacrimal drainage system stenosis (Munk grade ≥3), including both nasolacrimal duct (NLD) stenosis and common canalicular stenosis, were recruited in this prospective comparative case series. Patients underwent probing and either Monoka (51 eyes) or Masterka (48 eyes) intubation under general or local anesthesia. Tubes were removed 4–14 weeks after the procedure. Six months after tube removal, Munk grades 0 and 1 were defined as a complete success, Munk grade 2 was defined as a partial success, and Munk grade ≥3 was defined as failure. All complications were recorded.

**Results:**

Ninety-nine eyes from 89 patients with lacrimal drainage system stenosis who underwent either Monoka (51 eyes) or Masterka (48 eyes) intubation were included. The mean (SD) age of the patients was 55.4 (12) years in the Monoka group and 53.5 (12.9) in the Masterka group. Groups were matched on demographics. Masterka intubation could not be performed in one eye. Complete and partial successes were observed in 52.9% (27/51) and 17.6% (9/51) of eyes in the Monoka group and 42.6% (20/47) and 12.8% (6/47) of eyes in the Masterka group, respectively (*p*=0.29). There was a trend toward a higher total success rate in patients with NLD stenosis treated with Monoka 66.7% (26/39) than Masterka 45.5% (15/33) intubation (*p*=0.07). This trend also existed in patients with common canalicular stenosis (83.3% (10/12) vs. 76.6% (11/14), *p*=0.75). Age, sex, bilateral involvement, and duration of intubation did not have a significant impact on the success rate. Early tube loss, slit puncta, and temporary superficial punctate keratopathy were observed complications.

**Conclusion:**

Intubation with the pulled monocanalicular silicone tube was associated with a slightly but not significantly higher success rate in adults with lacrimal drainage system stenosis. Patients with NLD stenosis may achieve better results with pulled silicone tubes.

## 1. Introduction

Incomplete lacrimal drainage system obstruction or lacrimal drainage system stenosis (LDSS) is considered when a patient present with epiphora shows simultaneous partial passage and partial reflux during lacrimal drainage system syringing [1, 2]. Various treatment options, including dacryocystorhinostomy (DCR) [3–5], balloon catheter dilatation [6, 7], and silicone intubation [8–13], have been reported to address the LDSS in adults. Nasolacrimal duct intubation is commonly performed in patients with LDSS using either bicanalicular or monocanalicular silicone tubes with comparable efficacy results [10–13]. Compared to bicanalicular silicone tubes, monocanalicular intubation could be potentially less traumatic to the lacrimal system and do not require an additional procedure for its removal [14, 15].

Monocanalicular silicone tubes are commonly inserted through pulling from the nose (Monoka; FCI, Paris, France), in which nasal manipulation is mandatory. A new monocanalicular silicone tube with a pushed stent and self-retaining metallic fixation (Masterka; FCI, Marshfield Hills, MA, USA) was introduced. Masterka could be inserted by pushing from punctum-canaliculus with a relatively simple technique [16–18]. Pushed monocanalicular intubation has been reported to be effective in congenital nasolacrimal duct (NLD) obstruction [16, 17]. However, to our knowledge, there is no study comparing two types of monocanalicular intubation in adults with LDSS. The aim of this study was to compare the efficacy of and complications of Monoka (pulled) versus Masterka (pushed) monocanalicular silicone tubes in adults with LDSS concerning their chief compliant, epiphora.

## 2. Materials and Methods

This prospective institutional interventional comparative case series was conducted between November 2012 and March 2015 at Farabi Hospital. The Ethics Board Committee of Tehran University of Medical Science approved the study protocol. Informed consent was obtained from all participants.

Patients with acquired symptomatic epiphora were evaluated. The severity of epiphora was graded based on the Munk grading system (Table 1) [19].

Slit-lamp biomicroscopy and lacrimal drainage system evaluations, including the dye disappearance test, probing of the upper lacrimal drainage system, and syringing, were performed in all patients. The fluorescein dye disappearance test was performed by instilling a drop of 2% fluorescein in the conjunctival cul-de-sac and assessing the tear meniscus after 5 minutes. Patients with LDSS defined as a history of tearing (Munk grade ≥3) for at least 3 months, positive 5-minute fluorescein dye disappearance test, and simultaneous reflux through the opposing punctum and drainage into the nose on diagnostic irrigation of the lacrimal drainage system were enrolled. Feeling a “soft stop” on diagnostic probing signified CC stenosis; otherwise, a normal diagnostic probing and existence of LDSS criteria implicated NLD stenosis. Exclusion criteria were as follows: the previous lacrimal system or nasal surgery and or trauma, complete obstruction of the lacrimal drainage system, punctual or proximal canalicular stenosis, less than 18 years of age, ocular surface disease, eyelid malposition, blinking problem, any history of congenital NLD obstruction or stenosis, and less than 6 months of follow-up. Patients in whom tubes could not be passed through the NLD or fixed were excluded from the analysis.

Patients were nonrandomly allocated to either pulled (Monoka; FCI, Paris, France) or pushed (Masterka; FCI, Marshfield Hills, MA, USA) monocanalicular intubation. Monoka (3 mm flange) tubes were inserted under general anesthesia with the previously described technique (11). All tubes were easily retrieved, and no nasal endoscopy was performed. Intubation with a 40 mm Masterka was performed under general or local anesthesia (tetracaine 0.5%). The nasal endoscopy was performed for the 3 first cases to see if the tube reaches the inferior turbinate after punctum dilation using a punctum dilator. A metal probe was pulled out, while the punctal plug was held with a forceps. Both Monoka and Masterka tubes were fixed in the lower punctum. All procedures were performed by the same oculoplastic surgeon (B. E). Topical chloramphenicol 0.5% and betamethasone 0.1% eye drops were prescribed 4 times a day for 1 week. Silicone tube removal was scheduled in outpatient visit at 6–8 weeks postoperatively. Patients were also assessed 6 months after tube removal to assess the subjective success of the procedure by the Munk score.

Success was defined based on subjective success based on Munk grading 6 months after silicone tube removal. Lacrimal drainage system irrigation was not performed. Complete success was defined as a Munk grade of 0 and 1, partial success was defined as a Munk grade of 2, and failure was defined as a Munk grade of ≥3. Total success was considered if complete or partial success was achieved.

All data were collected at baseline and after 6 months of tube removal. Student's *t*-test and chi-square/Fisher's exact test were used to compare variables between groups. Statistical analysis was performed using SPSS software for Windows version 16 (SPSS Inc., Chicago, IL, USA). A *p* value less than 0.05 was considered statistically significant.

## 3. Results

Ninety-nine eyes from 89 patients with LDSS who underwent Monoka (51 eyes) or Masterka (48 eyes) intubation were included. Masterka silicone tube was unintentionally withdrawn in an accordion-like fashion during metal probe extraction in one case, and the patient was removed from the analysis. Surgical insertion was successful in all cases of Monoka intubation. Age, sex, laterality, site of stenosis, and preoperative Munk score were not significantly different in the two groups (Table 2, all variables *p* > 0.05).

Although tube removal was scheduled to perform at 6–8 weeks postoperatively, average removal times ranged between 4 and 14 months. There was also no difference in time to tube removal between groups (8.7 ± 1.6 and 8.3 ± 1.4 weeks in Monoka and Masterka group, respectively, *p*=0.36).

Complete success was achieved in 27 of 51 eyes (52.9%) in the Monoka group and 20 of 47 eyes (42.6%) in the Masterka group. Partial success was achieved in 9 of 51 eyes (17.6%) in Monoka group and 6 of 47 eyes (12.8%) in the Masterka group. Two success rates were not significantly different between both groups (*p*=0.16). The total success rate based on the level of stenosis in each group is illustrated in Table 3.

There was a trend toward a higher success rate in patients with NLD stenosis treated with Monoka 66.7% (26/39) than Masterka 45.5% (15/33) intubation (*p*=0.07). In the eyes with CC stenosis, the total success rate was relatively similar in Monoka and Masterka groups (83.3% (10/12) vs. 76.6% (11/14) respectively, *p*=0.75).

Clinical and demographic characteristics in patients with or without total successful monocanalicular intubation are compared as given in Table 4.

Age, sex, time of tube removal, bilateral involvement, and presence of complications did not differ between total success and failure groups. In the Masterka group, patients with CC stenosis showed a significantly higher success rate than patients with NLD stenosis (*p*=0.04).

Early tube loss (less than six weeks) occurred in one eye in the Monoka group and two eyes in the Masterka group all in week 4. Tube-related complications included superficial punctate keratopathy and slit punctum. Superficial punctate keratopathy was observed in 4 eyes with the Monoka tube (two cases in week 6 and two cases in week 8) and 7 eyes with the Masterka tube (two cases in week 6 and five cases in week 8), which resolved within one week after tube removal in all patients. Slit punctum occurred in 2 eyes with a Masterka tube that was observed in week 6.

## 4. Discussion

Lacrimal drainage system intubation has been widely proposed as the primary intervention in managing LDSS stenosis in adults [2, 9–11]. Although based on Poiseuille's law, lacrimal system intubation with bicanalicular tubes reduces the outflow resistance much more than monocanalicular tubes [14]; bicanalicular intubation is more likely to induce trauma to the lacrimal drainage system during insertion, impose more difficulty for removal, and holds the same success rate as monocanalicular intubation [10, 11] in patients with NLD stenosis.

Considering that monocanalicular intubation compared to bicanalicular is equally effective and easier to perform with less trauma, we decided to compare the two types of monocanalicular intubation in patients with LDSS.

In our study, monocanalicular intubation in adults with LDSS using Monoka and Masterka monocanalicular stents resulted in 70.6% and 55.3% total success rates during 6 months of follow-up, respectively. Our results are comparable with previous investigations regarding bicanalicular or monocanalicular intubation in adults. Recently, pushed monocanalicular silicone stents have been proven effective. Silicone tube intubation in adults was initially introduced by Keith [20] with 73% success rate using the bicanalicular tube. Connell et al. [9] studied long-term results of bicanalicular intubation for CC or NLD stenosis in 65 eyes. They reported a complete success of 50.7% and a partial success of 38.5%, with a mean follow-up of 78 months. In another study, Andalib et al. [10] found a success rate of 76% using either bicanalicular or pulled monocanalicular silicone tubes.

Recently, pushed monocanalicular silicone tubes have been proved effective in congenital NLD obstruction [18, 21–25], but there is a lack of investigations evaluating its utility in adults [23]. In this study, we used pushed monocanalicular silicone tube (Masterka) to manage LDSS in adults for the first time. Our results showed that Masterka intubation resulted in a lower success rate than Monoka intubation in adults with LDSS, although the difference was not statistically significant. Monoka has a slightly lower diameter than Masterka (0.64 mm vs. 0.90 mm), so we hypothesized that the lower compression on the stenotic nasolacrimal duct might induce lower following fibrotic reaction and is related to higher success. Probing and intubation is an acceptable method for NLDO in pediatric patients. Andalib and Mansouri's studied on children [24]. They showed that pushed monocanalicular silicone tube intubation has a lower success rate than pulled tubes in congenital NLD stenosis. Elewah et al. also reported a higher success rate with pulled tubes than pushed monocanalicular intubation in children aged 12–48 months [26].

On the other hand, in Vernat-Tabarly's study, the pushed method was insignificantly more successful than the pulled method [24]. But it should be noted that age is negatively correlated with silicone tube intubation success in lacrimal drainage system stenosis [27]. Our findings agree with the use of this method in adults suffering LDSS.

According to our results, pushed silicone tubes were less effective than the pulled ones in patients with adult NLD stenosis. Furthermore, intubation with Masterka could not be possible in one case due to tube extraction during metal probe removal. Although intubation with pushed stents may bring several advantages over pulled stenting, including simpler insertion technique, less nasal trauma, and the ability to perform under local anesthesia [16–18, 23], it may be associated with a lower success rate, especially in adults.

Intubation with both pulled and pushed silicone tubes showed higher efficacy in CC stenosis than NLD stenosis, but the difference was significant only in the Masterka group. In consistent with our findings, Connell et al. [9] have shown that bicanalicular intubation resulted in a higher success rate in adults with common canalicular involvement, but they made no distinction between complete obstruction and partial stenosis. In contrast, in Kashkouli' study, the presence or absence of associated CC membranous stenosis did not significantly change the success rate of silicone tube intubation [11].

Like some other studies [11, 14], the silicone tube was planned to be removed 6–8 weeks after the intubation. It seems that complications of silicone intubation mostly occurred after the second month of tube insertion [28]. In our study, all complications occurred after 4 weeks of intubation. The mean time of tube removal was not significantly different between two groups. Premature tube extrusion, temporary corneal epithelial erosion, and slit punctum were found in few eyes, which did not affect the success rate in either group.

One limitation of this study could be the absence of objective assessment of success through the irrigation test. We only evaluated subjective success. Furthermore, any further treatment would be based on patients' symptoms rather than the irrigation test. In addition, the sample size was not calculated in advance, which could especially bias the insignificant differences in the analysis. Therefore, no significant difference in the success rate between two groups may be due to the low number of included patients. A discrepancy between the range of tube removal time and scheduled removal time was another limitation of this study, although this factor was not significantly different between the two groups. In conclusion, pulled monocanalicular intubation resulted in slightly higher success than pushed ones in adults with LDSS.

## Figures and Tables

**Table 1:** Munk score.

Munk scale for epiphora grading [19]	
0	No epiphora
1	Occasional epiphora requiring drying or dabbing less than twice daily
2	Epiphora requiring dabbing 2–4 times daily
3	Epiphora requiring dabbing 5–10 times daily
4	Epiphora requiring dabbing more than 10 times daily or constant epiphora

**Table 2:** Comparison of demographic and clinical characteristics between different monocanalicular tube groups.

Variables	Monoka (51 eyes from 48 patients)	Masterka (47 eyes from 41 patients)	*P* value^*∗*^
Mean age (SD) (years)	55.4 (12)	53.5 (12.9)	0.42
Median age (range) (years)	58 (25–71)	55 (26–72)	

Sex (male/female)	18/30	18/23	0.54

Laterality (right/Left)	30/21	26/21	0.72

Bilateral involvement	3/48 (6.2%)	6/41 (14.6%)	0.30

Munk grade 3	36/51 (70.6%)	27/47 (57.4%)	0.17
Munk grade 4	15/51 (29.4%)	20/47 (42.6%)	

Level of stenosis (NLD)	39/51 (76.5%)	33/47 (70.2%)	0.48
Level of stenosis (CC)	12/51 (23.5%)	14/47 (29.8%)	

Mean tube removal time (SD) (weeks)	8.7 (1.7)	8.4 (1.5)	0.36
Median tube removal time (range) (weeks)	8 (4–14)	8 (4–13)	

**Table 3:** Comparison of total success in Monoka and Masterka groups based on the level of stenosis.

		Monoka (51 eyes)	Masterka (47 eyes)	*P* value
Nasolacrimal duct	Total success	26/39 (66.7%)	15/33 (45.5%)	0.07
Common canaliculus	Total success	10/12 (83.3%)	11/14 (76.6%)	0.75
Total	Total success	36/51 (70.6%)	26/47 (55.3%)	0.12

**Table 4:** Comparison of clinical and demographic characteristics in patients with or without total successful monocanalicular intubation.

Variables	Monoka (51 eyes from 48 patients)		*P* value	Masterka (47 eyes from 41 patients)		*P* value
	Total success	Failure		Total success	Failure	
Mean age (SD) (years)	56.3 (12.5)	53.1 (10.8)	0.37	55.1 (11.9)	51.6 (14)	0.36

Gender (male)	13/18 (72.2%)	5/18 (27.6%)	0.87	9/18 (50%)	9/18 (50%)	0.47
Gender (female)	21/30 (70%)	9/30 (30%)		14/23 (60.9%)	9/23 (39.1%)	

Level of stenosis (NLD)	26/39 (66.7%)	13/39 (33.3%)	0.31	15/33 (45.5%)	18/33 (54.5%)	0.04
Level of stenosis (CC)	10/12 (83.3%)	2/12 (16.7%)		11/14 (76.6%)	3/14 (21.4%)	

Mean tube removal time (SD) (weeks)	8.9 (1.6)	8.5 (1.9)	0.45	8.6 (1.5)	8.2 (1.4)	0.43

Bilateral involvement	4/6 (66.7%)	2/6 (33.3%)	0.93	6/12 (50%)	6/12 (50%)	0.67

Complications	3/5 (60%)	2/5 (40%)	0.62	7/11 (63.6%)	4/11 (36.4%)	0.73

## Data Availability

The data used to support the findings of this study are available from the corresponding author upon request.

## Abbreviations

NLD: Nasolacrimal duct
LDSS: Lacrimal drainage system stenosis
DCR: Dacryocystorhinostomy
CC: Common canaliculus.
